# Impact of *Helicobacter pylori* Virulence Genotypes *cagA*, *vacA*, *oipA*, and *babA2* on Severity of Gastropathies in Brazilian Patients

**DOI:** 10.3390/ijms26199471

**Published:** 2025-09-27

**Authors:** Diogo Nery Maciel, Hellen Christina de Oliveira Santos-Dutra, Viviane Lopes Rocha, Lucas Trevizani Rasmussen, Mônica Santiago Barbosa

**Affiliations:** 1 Institute of Tropical Pathology and Public Health, Federal University of Goiás, Goiânia 74690-900, Brazil; diogomaciell@yahoo.com.br (D.N.M.); hellen_oliveira@discente.ufg.br (H.C.d.O.S.-D.); vivianelopes@ufg.br (V.L.R.); 2 Department of Biomedicine, University Center of Integrated Faculties of Ourinhos, Ourinhos 19909-100, Brazil; lucasrasmussen@gmail.com

**Keywords:** outer membrane adhesin, genotype combinations, gastroduodenal diseases

## Abstract

*Helicobacter pylori* (*H. pylori*) is a Gram-negative, spiral-shaped bacterium that colonizes the human stomach and is linked to various gastroduodenal diseases. The severity of different clinical outcomes may be determined by the combination of virulence genes. The aim of this study was to assess the combinations of the cytotoxin-associated gene A (*cagA*), the vacuolating cytotoxin A gene (*vacA*), the outer inflammatory protein A gene (*oipA*), and the blood group antigen-binding adhesin gene (*babA2*) genotypes in *H. pylori* and their associations with the clinical outcomes of infection in patients from Central Brazil. This cross-sectional study included 106 patients who underwent endoscopy or gastrectomy. The presence and genotypes of *H. pylori* were confirmed using Polymerase Chain Reaction (PCR). Gastropathies were classified according to established severity criteria. Multivariate logistic regression and Venn diagrams were used to evaluate gene combinations. In this study, the infection prevalence was 65.1%. The *cagA*/*vacA*/*oipA*/*babA2* combination showed a protective effect against erosive esophagitis (*p* = 0.002), erosive duodenitis (*p* = 0.003), and general duodenitis (*p* < 0.001). No significant association was observed between this gene combination and severe gastric diseases, although a trend toward protection against gastric atrophy was noted (*p* = 0.049). These findings suggest that the coexistence of *cagA*/*vacA*/*oipA*/*babA2* may play a protective role against inflammatory lesions. Further studies should explore the functional role of these gene combinations, also considering the immunogenetic profile of the host.

## 1. Introduction

*Helicobacter pylori* (*H. pylori*) harbors a range of virulence genes that are associated with various gastric pathologies, including gastritis, peptic ulcers, Mucosa-Associated Lymphoid Tissue (MALT) lymphoma, and gastric cancer [1,2]. It is estimated that more than 50% of the global population is infected. However, prevalence rates vary substantially, as factors such as lifestyle and socioeconomic status directly influence the infection rate, which varies across different settings, being significantly higher in developing countries (50.8%), such as Brazil, compared to developed countries (34.7%) [1,3,4].

The infection often persists asymptomatically for long periods, which favors the progression of these gastropathies. It is a Gram-negative, microaerophilic, flagellated bacterium, whose adaptive capacity helps in the colonization of the human gastrointestinal tract of approximately half of the global population [5,6,7]. This successful adaptation of the bacterium to the hostile environment of the stomach is favored by a series of virulence factors, which allow its adhesion to the gastric epithelium and evasion of the host’s immune system [8,9].

Prevalence studies conducted in latin american populations have found rates between 60% and 91% [10,11,12]. The choice of the central-west region of Brazil for this study was due to the geographic diversity of the pathogen and the scarcity of data related to the molecular epidemiology of *H. pylori* in this area. This region is characterized by significant socioeconomic contrasts, the coexistence of urban and rural areas with different levels of infrastructure and access to health care, and weaknesses in epidemiological surveillance. It is believed that these factors collectively influence both the prevalence of infection and the genetic variability of circulating strains, highlighting the importance of conducting specific regional studies.

Among the most studied virulence factors of *H. pylori* are the *cagA*, *vacA*, *oipA*, and *babA* genes, which play fundamental roles in the pathogenesis of the infection. The CagA (cytotoxin-associated gene A) protein is one of the main proteins responsible for inducing inflammatory responses and altering cellular signaling in the host. Its activity, particularly in association with the type IV secretion system, is strongly implicated in the genesis of epithelial lesions and gastric carcinogenesis [13]. VacA (vacuolating cytotoxin A) plays an important role in modulating the immune response and vacuolation of gastric cells, inducing cellular damage and stimulating an environment conducive to pathogen persistence [14].

The OipA (outer Inflammatory Protein A) plays a key role in modulating gastric inflammation by inducing the expression of pro-inflammatory cytokines, like interleukin-8 (IL-8), and enhancing bacterial adhesion to the stomach mucosa, which exacerbates the inflammatory response [15]. OipA expression triggers apoptotic pathways and cytoskeletal changes in gastric epithelial cells, contributing to the progression of severe gastropathies [16]. On the other hand, the BabA protein (blood-group antigen-binding adhesin) acts as a specialized adhesin, mediating the attachment of *H. pylori* to glycosylated structures expressed on the gastric mucosa. This interaction is a critical step in establishing and maintaining the host-pathogen relationship [17].

*H. pylori*’s extensive genetic plasticity influences the clinical variability observed in infected individuals. Co-expression of certain genes can amplify inflammation, promote infection persistence, and increase the risk of severe gastric complications [18]. Studies indicate that the association between certain alleles of cytotoxin-associated gene A (*cagA*) and vacuolating cytotoxin A gene (*vacA*), for example, is directly related to the progression to gastric cancer, while the concomitant expression of outer inflammatory protein A gene (*oipA*), and the blood group antigen-binding adhesin gene (*babA2*) may confer a greater capacity for adhesion and colonization of the gastric epithelium [19,20].

Recent research indicates that the expression of OipA is associated with other *H. pylori* virulence markers, such as *cagA*, *vacA s1*/*m1* and *babA2*. In strains with preserved *cag*-PAI pathogenicity island, there is greater coexpression of *oipA*, which suggests a possible synergistic effect between these genetic determinants. The geographic variability of these virulence factors may represent an adaptive mechanism to the gastric microenvironment, which interacts with host elements and environmental factors, and influences the clinical manifestations of the infection [2,21].

Therefore, the combination of multiple virulence factors plays a critical role in the progression of infection and the severity of associated gastric lesions. Integrated analysis of these genetic profiles is essential for understanding the pathogenesis of *H. pylori* [15].

The advent of multi-omic approaches, integrating genomics, transcriptomics, proteomics, and metabolomics, has decisively expanded the understanding of the *Helicobacter pylori*–host relationship. The simultaneous analysis of multiple molecular contexts can yield consistent associations between virulence factors and clinical outcomes [22,23,24]. Within this framework, the present study employs a combinatorial gene analysis approach, aligning with contemporary trends in systems biology to provide an integrated perspective on *H. pylori* virulence.

This study aimed to investigate the combinations between the *cagA*, *vacA*, *oipA*, and *babA2* genotypes of *H. pylori* and their associations with different clinical outcomes in patients from Central Brazil.

## 2. Results

### 2.1. Demographic and Clinical Characteristics of Participants

A total of 106 patients with dyspepsia, who underwent upper gastrointestinal endoscopy or gastrectomy as indicated for diagnostic purposes, were included in the study. Of these, 69 tested positive for *H. pylori*, representing an approximate prevalence of 65.1% in this sample. The population consisted of 82 females and 24 males, aged between 18 and 75 years, with a predominance of individuals over 51 years of age. Infection was more common among participants with primary education and those from families with a monthly income between one and two minimum wages (Table 1).

### 2.2. Endoscopic Findings and Their Association with H. pylori Infection

Table 2 presents the distribution of clinical–endoscopic findings in the sample of 106 patients with dyspepsia, classified according to the presence or absence of *H. pylori*. Among the infected patients (*n* = 69), the most common findings were anthematous gastritis (42.0%) and erosive gastritis (30.4%). Less prevalent findings included gastric adenocarcinoma (2.9%), intestinal metaplasia (2.9%), and gastric ulcer (1.4%). In the non-infected group (*n* = 37), anthematous gastritis was also the most common finding (37.8%), followed by erosive gastritis (29.7%) and gastric atrophy (18.9%). A significant difference was observed for the diagnosis of gastric atrophy (*p* = 0.035), which was more prevalent among non-infected patients. Other diagnoses, such as intestinal metaplasia, erosive duodenitis, erosive esophagitis, and general gastritis, did not show statistically significant differences between the groups (*p* > 0.05). The overall frequency of severe gastric disease was relatively low in both groups, with a predominance of non-severe lesions in the total sample.

The distribution of *H. pylori* virulence gene combinations according to endoscopic diagnosis is described in Table 3.

### 2.3. Association Between Virulence Genes and Demographic or Clinical Variables

The *cagA*/*babA2* combination had the highest overall frequency (21.7%), followed by *cagA*/*oipA* (13.0%) and *cagA*/*oipA*/*babA2* (10.1%). No gene combination showed a statistically significant association with the severity of gastric disease (*p* > 0.05). The analysis of *H. pylori* virulence gene combinations is essential for understanding the impact of these genetic variants on the pathogenesis of gastric diseases. However, the lack of significant association with clinical outcomes observed in this study may reflect the complexity of interactions among microbial virulence factors, host genetics, and other factors that influence disease progression.

The *cagA*/*vacA*/*oipA*/*babA2* gene combination did not show significant differences regarding age group (*p* = 0.120) or sex (*p* = 0.100), despite the predominance of this gene combination in the female group (80%). The association with gastric adenocarcinoma and intestinal metaplasia was not statistically significant, but a protective trend against gastric atrophy was observed (*p* = 0.049; Odds Ratio (OR) = 0.88; 95% Confidence Interval (CI): 0.75–0.98). Among non-severe diseases, the presence of this gene combination was correlated with a lower risk of erosive duodenitis (*p* = 0.003; OR = 0.65; 95% CI: 0.49–0.86) and erosive esophagitis (*p* = 0.002; OR = 0.60; 95% CI: 0.42–0.81). Additionally, individuals with the *cagA*/*vacA*/*oipA*/*babA2* combination had a lower likelihood of developing general duodenitis (*p* < 0.001; OR = 0.54; 95% CI: 0.39–0.76). These findings suggest a potential protective role for this gene combination against inflammatory lesions in the gastrointestinal tract (Table 4).

### 2.4. Distribution Patterns of Virulence Gene Combinations

**Figure 1:**
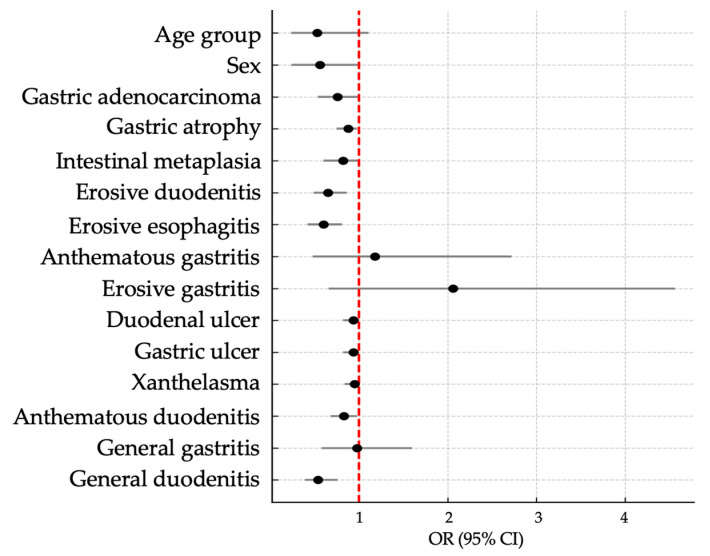
A Forest Plot demonstrating the Odds Ratios for the association of the *cagA*/*vacA*/*oipA*/*babA2* gene combination with sociodemographic variables and gastropathies. The horizontal lines represent the 95% confidence intervals (CI) of the Odds Ratio (OR) estimates, while the black dots indicate the estimated point values for each group or variable. The vertical red dashed line marks the null value (OR = 1), which indicates no statistical association between the analyzed variable and the outcome.

A more detailed analysis of the *cagA*/*vacA*/*oipA*/*babA2* gene combination and predictive variables, such as sociodemographic characteristics, risk factors, and the severity of gastropathies, is presented in the Forest Plot shown in Figure 1, which displays the Odds Ratio values.

**Figure 2:**
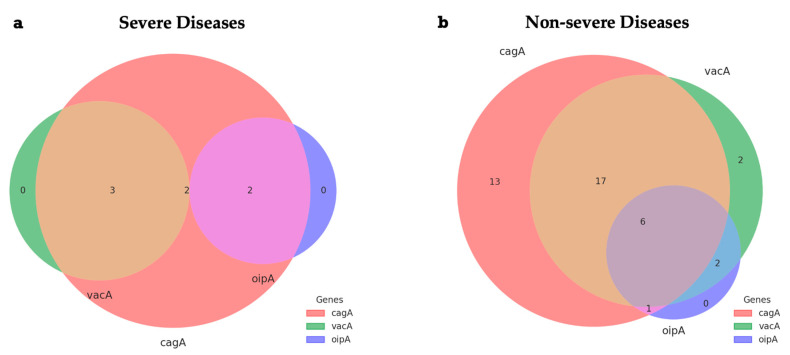
Venn diagrams representing the distribution of *H. pylori* virulence gene combinations (*cagA*, *vacA*, and *oipA*) in samples from patients with severe (**a**) and non-severe (**b**) gastric diseases. Each color represents the presence of a gene or gene combination (salmon: *cagA*; green: *vacA*; purple: *oipA*; lilac: overlap between *cagA* and *oipA*; lilac–gray: overlap between *cagA*, *vacA*, and *oipA*; blue–green: overlap between *oipA* and *vacA*; brown: overlap between the *cagA* and *vacA* genes).

The Venn diagrams represented in Figure 2 show the distribution of combinations of the *H. pylori* virulence genes *cagA*, *vacA*, and *oipA* in patients diagnosed with severe or non-severe gastric disease. The intersections indicate the coexistence of two or more genes in the same sample. In the group with severe disease, seven samples exhibited the *cagA* gene, of which three had *vacA* coexisting, two had *oipA* coexisting, and two had *cagA*, *vacA*, and *oipA* simultaneously. The isolated presence of *vacA* or *oipA* was not observed in this group.

**Figure 3:**
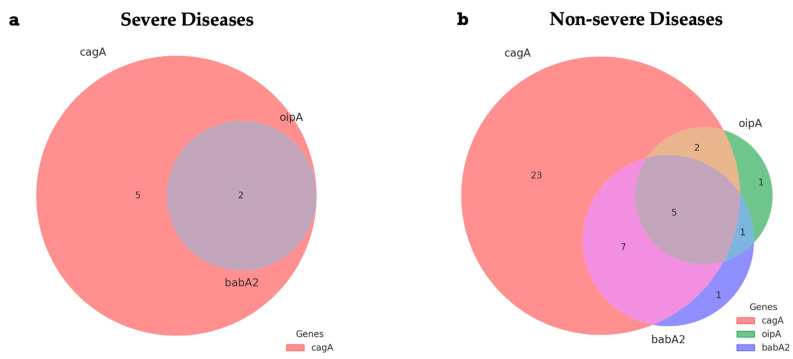
Venn diagrams representing the intersections of *H. pylori* virulence gene combinations (*cagA*, *oipA*, and *babA2*) in samples from patients with severe (**a**) or non-severe (**b**) gastric diseases. Each color represents the presence of a gene or gene combination (salmon: *cagA*; green: *oipA*; purple: *babA2*; lilac–gray: overlap between *cagA*, *oipA*, and *babA2*; lilac: overlap between *cagA* and *babA2*; blue–green: overlap between *oipA* and *babA2*; brown: overlap between the *cagA* and *oipA* genes).

Figure 3 shows two Venn diagrams that illustrate the distribution of combinations of the *H. pylori* virulence genes *cagA*, *oipA*, and *babA2* in patients diagnosed with severe or non-severe gastric disease. The intersections indicate the coexistence of two or more genes in the same sample. Among the cases classified as severe, five samples presented only the *cagA* gene, while two samples simultaneously exhibited *cagA*, *oipA*, and *babA2*. No cases of coexistence between *cagA* and *babA2*, or isolated manifestation of *oipA* or *babA2*, were identified in this group. In the group with non-severe disease, the predominance of samples positive only for *cagA* was observed (*n* = 23). In addition, seven samples presented coexistence of *cagA* and *babA2*, five simultaneously exhibited the three genes (*cagA*, *oipA*, and *babA2*), and two cases were positive for *cagA* and *oipA*. Three unique observations were also detected: one for *oipA*, one for *babA2*, and a combination of *oipA* and *babA2* without *cagA*.

## 3. Discussion

This study evaluated the combinations of the *cagA*, *vacA*, *oipA*, and *babA2* virulence genes and their associations with the severity of gastrointestinal lesions in a dyspeptic population infected with *H. pylori* in the Central region of Brazil. The prevalence of infection was 65.1. The distribution of the studied genes and sociodemographic variables, such as age and sex, did not show significant differences. These findings support observations that the pathogenicity of *H. pylori* seems to be more related to bacterial and immunological factors than to isolated sociodemographic factors [6,25].

The frequency of the *cagA* (79.7%) and *vacA* (53.6%) genes was higher in this study, followed by *babA2* (15.9%) and *oipA* (26.1%). Although *cagA* was more common among cases of severe disease, its presence did not reach statistical significance, similar to the other genes. Previous studies suggest that the isolated presence of these genes does not absolutely predict the severity of clinical manifestations, but certain combinations can enhance the microorganism’s infection capability [26,27].

In this study, the *cagA*/*vacA*/*oipA*/*babA2* combination showed a significant protective association against inflammatory lesions, such as erosive duodenitis, erosive esophagitis, and general duodenitis. These findings support the idea that the simultaneous interaction of multiple virulence factors can modulate the host’s inflammatory response, reducing tissue damage [28]. Despite the low frequency of subgroups expressing the *cagA*/*vacA*/*oipA*/*babA2* gene combination, the complete absence of certain inflammatory lesions among these individuals suggests a potential factor associated with clinical protection. Although numerically limited, the asymmetric distribution led to statistically significant associations, raising the hypothesis of a possible biological effect.

The combined analysis of the *cagA*, *vacA*, *oipA*, and *babA2* genes in this study was based on both their functional roles, described in the literature, and the frequency with which these genes are investigated as key *H. pylori* virulence markers. While some studies have not found a direct association between these genes and specific clinical outcomes [29,30], others have suggested trends within subgroups, such as the significant association observed for the *cagA*+/*vacA* s1/m1 genotype [31]. Furthermore, Barhoine et al. (2025) [2] reported that although co-occurrence among several virulence genes was statistically significant in their study, *oipA*, *babA*, and *iceA* showed no direct correlation with histopathological parameters, reinforcing the complexity of gene–gene interactions and the importance of contextual analysis.

This complex interaction between pathogen and host mechanisms, reinforced by the combination of virulence genes, allows for successful bacterial colonization through evasion of the immune system [32].

Our results suggest that the combined presence of the *cagA*, *vacA*, and *oipA* genes is more common in non-severe forms of the disease, although the *cagA* gene is present in all cases classified as severe. This contrasts with a study conducted in the Southeast region of Brazil, where the combination of the *cagA*, *vacA*, *dupA*, and *oipA* virulence factors showed high prevalence in patients with chronic gastritis and gastric cancer [31].

The absence of *vacA* and *oipA* as isolated factors in severe diseases reinforces the possibility that increased pathogenicity is more associated with the presence of *cagA* or the combination of multiple genes. In contrast, non-severe lesions demonstrated greater diversity of gene combinations, while severe disease cases showed a more restricted profile, concentrated on the isolated presence of *cagA* or the triple gene association. In a study conducted in Ecuador, the *cagA*/*oipA* combination was associated with an increased risk of developing acute gastric inflammation and lymphoid follicular hyperplasia [24].

The findings of this research reinforce the central role of the *cagA* gene in the virulence of the microorganism, especially in more aggressive clinical forms. The *cag* pathogenicity island (*cag*-PAI) contains genes encoding a secreted effector protein (CagA) and components of a type IV secretion system (Cag T4SS), which are closely related to the pathogenesis of *H. pylori* [33]. Approximately 60% to 70% of *H. pylori* strains express CagA, leading to increased synthesis of interleukin-8 (IL-8). The presence of tyrosine-containing motifs in the CagA structure, once phosphorylated, allows for its translocation into gastric epithelial cells via the Cag T4SS. Alterations in the sequence of these motifs have been correlated with gastric mucosal degeneration and increased predisposition to gastric adenocarcinoma [34].

The *oipA* and *babA2* genes, important for bacterial adhesion to the gastric mucosa, showed prevalences of 15.9% and 26.1%, respectively. Despite their recognized role in bacterial adhesion and inflammation induction, their isolated combinations were not associated with lesion severity, suggesting that their influence depends on the genetic context of the strain and host factors. The lower frequency of the *oipA* genotype observed in this study contrasts with data from populations in Vietnam, where high prevalence and association with pre-neoplastic gastric mucosal alterations have been reported [12]. Conversely, despite the high detection of *oipA* in studies conducted in China, no significant correlation was found between this genotype and gastroduodenal clinical manifestations [14]. These data may reflect unique characteristics of the studied populations.

Among the main limitations of this study are the exploratory design, including the reduced sample size of specific subgroups, especially the subgroup with severe clinical manifestations, in addition to the absence of functional analyses and the lack of control for host variables. However, the findings reinforce the importance of the molecular characterization of *H. pylori* strains and serve as a basis for future hypotheses that encourage the development of broader genomic and immunogenetic approaches, with more integrative investigative strategies. Its contribution lies in the identification of preliminary patterns that should be validated by future studies with greater sample representation and statistical power.

## 4. Materials and Methods

### 4.1. Population and Samples

This cross-sectional study included 106 individuals with dyspeptic symptoms, aged 18 to 75, who underwent upper gastrointestinal endoscopy or gastrectomy at referral hospitals in the city of Goiânia, Goiás, Brazil. The exclusion criteria ruled out patients currently using proton pump inhibitors, H2 receptor antagonists, immunosuppressive agents, or antibiotics. Additionally, participants with evidence of active gastrointestinal bleeding, pregnant or lactating women, and those deemed ineligible for endoscopy were excluded. All participants signed an informed consent form (ICF) and completed a sociodemographic questionnaire. The confidentiality and privacy of all personal data were strictly protected throughout the study, ensuring the integrity of participant information.

Sample collection followed the guidelines of the IV Brazilian Consensus on *Helicobacter pylori* Infection. During the endoscopic procedure, two biopsy samples were taken from the antral region and two from the gastric body, as recommended for histological representativeness [35]. Tumor samples from patients with gastric cancer were obtained during gastrectomy, ensuring the preservation of morphological integrity and neoplastic tissue representativeness for analysis [36].

One sample from the antrum and another from the gastric body were sent to the Clinical Pathology Laboratory of the University Hospital at the Federal University of Goiás (HC/UFG) for histopathological analysis. The two remaining fragments were refrigerated and stored at −20 °C in the *Helicobacter pylori* Research Center (NEHP) of the same institution. These samples were later subjected to molecular analysis following previously established protocols, as described in prior publications by the NEHP [37,38].

### 4.2. Histology

Gastric mucosa samples obtained through endoscopy or gastrectomy were fixed in 10% buffered formalin solution. The tissue fragments were then stained with hematoxylin-eosin and Giemsa. Histopathological evaluation was performed according to the Sydney System, which standardizes the classification of morphological changes in the gastric mucosa [39].

### 4.3. DNA Extraction

DNA extraction followed the manufacturer’s protocol for the QIAamp DNA Mini Kit^®^ (Qiagen, Valencia, CA, USA). The quantification and assessment of the purity of the extracted genomic DNA were conducted using optical density measurements with a NanoDrop^®^ ND-1000 UV-Vis spectrophotometer (Thermo Fisher Scientific, Wilmington, DE, USA), ensuring the integrity and quality of the genetic material for subsequent analyses.

### 4.4. DNA Amplification of H. pylori and Virulence Genes cagA, vacA, oipA, and babA2

The PCR technique was used for the molecular diagnosis of *H. pylori* infection, targeting the amplification of a fragment of the *16S ribosomal RNA* (*rRNA*) gene. Samples that tested positive for *H. pylori* were then further analyzed for the detection of the virulence genes *cagA*, *vacA*, *oipA*, and *babA2*.

The amplification of the 16S rRNA, *cagA*, *vacA*, *oipA*, and *babA2* genes was carried out using a BioRad^®^ thermal cycler (Bio-Rad Laboratories, Inc., Hercules, CA, USA). Each PCR reaction contained the following components: 0.5 µL of Taq DNA polymerase (2.5 units), 5 µL of CoralLoad 10X PCR buffer (QIAamp, Qiagen) containing 1.5 mM MgCl_2_, 2 µL of dNTPs (2.5 mM), and 33.5 µL of milli-Q water. After mixing, 5 µL of DNA from each sample (150 ng) was added, bringing the total reaction volume to 50 µL. DNA from *H. pylori*-positive samples was used as a positive control in the PCR reactions. DNA-free water was used as a negative control to ensure the absence of contamination and validate the specificity of the amplification process.

Details regarding the oligonucleotides used for the amplification of each gene, along with the PCR conditions and expected fragment sizes, are provided in Table 5 [40,41,42,43].

The PCR products were stained with BlueGreen nucleic acid dye (Lab Biotechnology^®^ Co., Ltd., Beijing, China) and subjected to electrophoresis on a 1.5% agarose gel. The visualization of the fragments was performed under ultraviolet (UV) light using a transilluminator, confirming the presence and size of the generated amplicons.

### 4.5. Diagnosis of Gastroduodenal Diseases and Severity Criteria

The stratification of patients with gastroduodenal lesions, based on the degree of severity, was conducted by the gastroenterology services and clinical pathology laboratories of the recruiting hospitals. This classification relied on the analysis of endoscopic reports and histopathological examinations. Clinical outcomes were grouped into two categories: non-severe, including duodenitis, esophagitis, gastritis, xanthelasma, and ulcers; and severe, comprising gastric adenocarcinoma, gastric atrophy, and intestinal metaplasia [44].

### 4.6. Data Analysis

Descriptive statistics were used to characterize the demographic and clinical profiles of the patients. Categorical variables were expressed as absolute frequencies (*n*) and relative frequencies (%), while continuous variables were reported as means and standard deviations. To compare the distribution of patient characteristics according to *H. pylori* infection status (positive vs. negative), Pearson’s chi-square test was applied.

To investigate the associations of individual virulence genes (*cagA*, *vacA*, *dupA*, *oipA*, *babA2*) and their combinations (e.g., *cagA*/*vacA*/*oipA*/*babA2*, *cagA*/*oipA*, *cagA*/*babA2*, *cagA*/*oipA*/*babA2*) with clinical outcomes, multiple logistic regression models were fitted. Each model included one gene or gene combination as the binary outcome variable (presence/absence), and the clinical and demographic features were included as independent predictors. The predictor variables entered into the models were as follows: age group, sex, and the presence or absence of each of the following clinical diagnoses—gastric adenocarcinoma, gastric atrophy, intestinal metaplasia, erosive duodenitis, erosive esophagitis, enanthematous gastritis, erosive gastritis, duodenal ulcer, gastric ulcer, xanthelasma, enanthematous duodenitis, general gastritis, and general duodenitis.

To prevent multicollinearity and overfitting, all regression models were adjusted using ridge penalization. Odds Ratios, 95% Confidence Intervals, Wald statistics, and *p*-values were reported for each predictor variable.

Additionally, the distribution of virulence gene combinations was analyzed using binary coding (presence = 1, absence = 0), and patients were categorized into two clinical outcome groups, “Severe” and “Non-severe” gastropathy. Venn diagrams were used to graphically illustrate the intersections among gene combinations in both groups. These diagrams were created in R (version 12.1) using the *VennDiagram* and *ggVennDiagram* packages, applying the venn.diagram() function for clinical group stratification.

All statistical analyses were conducted using SPSS version 26.0 (IBM Corp., Armonk, NY, USA), with a two-tailed significance level set at α = 0.05.

## 5. Conclusions

In this study, the *cagA*/*vacA*/*oipA*/*babA2* combination demonstrated a potential protective effect against the occurrence of erosive esophagitis, erosive duodenitis, and unspecified duodenitis. These findings suggest that the synergistic interaction of multiple bacterial virulence determinants may modulate the host’s inflammatory response, directly influencing the clinical outcome of *H. pylori* infection. In exploratory studies involving low-prevalence genetic variables, such evidence may support initial hypotheses when interpreted with caution. In this context, the results, despite statistical limitations, may contribute to the understanding of the interactions between virulence genotypes and clinical manifestations of *H. pylori* infection. Future investigations should aim to further characterize virulence gene variants and explore the immunogenetic profiles of hosts, with the goal of elucidating the molecular mechanisms involved in pathogenesis and refining risk stratification for the development of more severe clinical complications.

## Figures and Tables

**Table 1:** The sociodemographic characteristics of the sample according to *H. pylori* detection (*n* = 106).

	Total *n* = 106	*H. pylori n* (%)		*p* *
		No *n* = 37	Yes *n* = 69	
**Age Group**				
<40 years	28 (26.4%)	5 (13.5%)	23 (33.3%)	0.087
40 to 50 years	19 (17.9%)	8 (21.6%)	11 (15.9%)	
51 years or more	59 (55.7%)	24 (64.9%)	35 (50.7%)	
**Sex**				
Female	82 (77.4%)	29 (78.4%)	53 (76.8%)	0.856
Male	24 (22.6%)	8 (21.6%)	16 (23.2%)	
Education Level **				
Illiterate	7 (6.6%)	3 (8.1%)	4 (5.8%)	0.463
Primary education	55 (51.9%)	20 (54.1%)	35 (50.7%)	
Secondary education	35 (33.0%)	13 (35.1%)	22 (31.9%)	
Higher education	9 (8.5%)	1 (2.7%)	8 (11.6%)	
**Monthly Family Income**				
<1 minimum wage	13 (12.3%)	3 (8.1%)	10 (14.5%)	0.471
1 to 2 minimum wages	36 (34.0%)	12 (32.4%)	24 (34.8%)	
3 to 5 minimum wages	33 (31.1%)	10 (27.0%)	23 (33.3%)	
5 or more minimum wages	4 (3.8%)	2 (5.4%)	2 (2.9%)	
Unknown	20 (18.9%)	10 (27.0%)	10 (14.5%)	

* Pearson’s chi-square; *n*, absolute frequency; %, relative frequency. ** Note: Education level was classified as follows: illiterate (no formal education), primary education (completed elementary school), secondary education (completed high school), and higher education (completed undergraduate or postgraduate studies), according to the Brazilian education system.

**Table 2:** The clinical profile of the sample according to *H. pylori* detection (*n* = 106).

	Total *n* = 106	*H. pylori n* (%)		*p* *
		No *n* = 37	Yes *n* = 69	
**Severe Diseases**				
Gastric adenocarcinoma	2 (1.9%)	0 (0.0%)	2 (2.9%)	0.296
Gastric atrophy	11 (10.4%)	7 (18.9%)	4 (5.8%)	0.035
Intestinal metaplasia	6 (5.7%)	4 (10.8%)	2 (2.9%)	0.093
**Non-severe Diseases**				
*Esophagus*				
Erosive esophagitis	15 (14.2%)	5 (13.5%)	10 (14.5%)	0.890
*Stomach*				
Anthematous gastritis	43 (40.6%)	14 (37.8%)	29 (42.0%)	0.675
Chronic gastritis	1 (0.9%)	1 (2.7%)	0 (0.0%)	0.170
Erosive gastritis	32 (30.2%)	11 (29.7%)	21 (30.4%)	0.940
Gastric ulcer	4 (3.8%)	3 (8.1%)	1 (1.4%)	0.086
General gastritis	74 (69.8%)	25 (67.6%)	49 (71.0%)	0.713
Xanthelasma (gastric)	1 (0.9%)	0 (0.0%)	1 (1.4%)	0.462
*Duodenum*				
Anthematous duodenitis	4 (3.8%)	0 (0.0%)	4 (5.8%)	0.135
Duodenal ulcer	1 (0.9%)	0 (0.0%)	1 (1.4%)	0.462
Duodenitis	1 (0.9%)	1 (2.7%)	0 (0.0%)	0.170
Erosive duodenitis	10 (9.4%)	2 (5.4%)	8 (11.6%)	0.299
General duodenitis	15 (14.2%)	3 (8.1%)	12 (17.4%)	0.191

* Pearson’s chi-square; *n*, absolute frequency; %, relative frequency.

**Table 3:** The characterization of genes and gene combinations according to the severity of gastropathies (*n* = 69).

	Total *n* = 69		Diagnostic		*p* *
		Normal 18 (17.0%)	Non-Severe 72 (67.9%)	Severe 16 (15.1%)	
**Genes**					
*cagA*	55 (79.7%)	11 (84.6%)	37 (75.5%)	7 (100.0%)	0.285
*vacA*	37 (53.6%)	7 (53.8%)	27 (55.1%)	3 (42.9%)	0.831
*oipA*	11 (15.9%)	0 (0.0%)	9 (18.4%)	2 (28.6%)	0.173
*babA2*	18 (26.1%)	2 (15.4%)	14 (28.6%)	2 (28.6%)	0.621
**Gene Combinations**					
*cagA*/*vacA*/*oipA*/*babA2*	5 (7.2%)	0 (0.0%)	5 (10.2%)	0 (0.0%)	0.333
*cagA*/*oipA*	9 (13.0%)	0 (0.0%)	7 (14.3%)	2 (28.6%)	0.173
*cagA*/*babA2*	15 (21.7%)	1 (7.7%)	12 (24.5%)	2 (28.6%)	0.383
*cagA*/*oipA*/*babA2*	7 (10.1%)	0 (0.0%)	5 (10.2%)	2 (28.6%)	0.130

* Pearson’s chi-square; *n*, absolute frequency; %, relative frequency.

**Table 4:** The distribution of the *cagA*/*vacA*/*oipA*/*babA2* gene combination and its association with sociodemographic variables and the severity of gastropathies.

	*cagA*/*vacA*/*oipA*/*babA2*		Wald	OR (95% CI)	*p* *
	Absent	Present			
**Age Group**					
18 to 50 years	31 (48.4%)	3 (60.0%)	2.42	Ref	
51 years or more	33 (51.6%)	2 (40.0%)		0.53 (0.24–1.11)	0.120
**Sex**					
Female	49 (76.6%)	4 (80.0%)	2.70	Ref	
Male	15 (23.4%)	1 (20.0%)		0.56 (0.24–1.02)	0.100
**Severe Diseases**					
Gastric adenocarcinoma	2 (3.1%)	0 (0.0%)	2.67	0.76 (0.54–1.00)	0.102
Gastric atrophy	4 (6.3%)	0 (0.0%)	3.63	0.88 (0.75–0.98)	0.049
Intestinal metaplasia	2 (3.1%)	0 (0.0%)	1.83	0.82 (0.60–1.00)	0.176
**Non-severe Diseases**					
*Esophagus*					
Erosive esophagitis	10 (15.6%)	0 (0.0%)	9.94	0.60 (0.42–0.81)	0.002
*Stomach*					
Anthematous gastritis	26 (40.6%)	3 (60.0%)	0.14	1.18 (0.48–2.72)	0.704
Erosive gastritis	18 (28.1%)	3 (60.0%)	2.26	2.06 (0.66–4.56)	0.133
Gastric ulcer	1 (1.6%)	0 (0.0%)	1.22	0.94 (0.82–1.00)	0.269
General gastritis	44 (68.8%)	5 (100.0%)	0.01	0.98 (0.58–1.60)	0.930
Xanthelasma (gastric)	1 (1.6%)	0 (0.0%)	1.07	0.95 (0.84–1.00)	0.302
*Duodenum*					
Anthematous duodenitis	4 (6.3%)	0 (0.0%)	3.81	0.83 (0.68–0.98)	0.049
Duodenal ulcer	1 (1.6%)	0 (0.0%)	1.22	0.94 (0.82–1.00)	0.269
Erosive duodenitis	8 (12.5%)	0 (0.0%)	9.11	0.65 (0.49–0.86)	0.003
General duodenitis	12 (18.8%)	0 (0.0%)	12.50	0.54 (0.39–0.76)	<0.001

* Multiple logistic regression; r^2^ = 0.21; CI = Confidence Interval; OR = Odds Ratio; Ref = reference; r^2^ = coefficient of determination.

**Table 5:** The genes and primer sequences used for *H. pylori* identification and genotyping, the amplification conditions, and the expected fragment sizes.

Gene	Sequence (5′ → 3′)	Amplification Conditions	bp
*16S* *rRNA*	CTGGAGARACTAAGYCCTCC GAGGAATACTCATTGCAAGGCGA	95 °C, 2 min; 95 °C, 1 min; 60 °C, 1 min; 72 °C, 1 min (40 cycles); 72 °C, 5 min [36]	150
*cagA*	ATGACTAACGAAACTATTGATC CAGGATTTTTGATCGCTTTATT	94 °C, 1 min; 53 °C, 1 min; 72 °C, 1 min (40 cycles) [37]	232
*vacA*	ATGGAAATACAACAAACACAC CCTGARACCGTTCCTACAGC	94 °C, 45 s; 54 °C, 45 s; 72 °C, 45 s (35 cycles) [39]	286
*oipA*	GTTTTTGATGCATGGGATTT GTGCATCTCTTATGGCTTT	94 °C, 1 min; 56 °C, 1 min; 72 °C, 1 min (35 cycles) [38]	401
*babA2*	CCAAACGAAACAAAAAGCGT GCTTGTGTAAAAGCCGTCGT	94 °C, 2 min; 94 °C, 45 s; 48 °C, 30 s; 72 °C, 60 s (35 cycles); 72 °C, 10 min [37]	271

°C = degrees Celsius, min = minutes, s = seconds.

## Data Availability

Additional data supporting the manuscript are available from the corresponding author upon request.

## Abbreviations

The following abbreviations are used in this manuscript:

*H. pylori*	*Helicobacter pylori*
*cagA*	Cytotoxin-associated A gene
*vacA*	Vacuolating cytotoxin A gene
*oipA*	Outer Inflammatory Protein A gene
*babA2*	Blood-group antigen-binding adhesin gene
*PCR*	Polymerase chain reaction
